# Research on the Secondary Forgeability of High Volume Fraction Whisker Reinforced Aluminum Matrix Composites of Original Squeeze Casting

**DOI:** 10.3390/ma14237261

**Published:** 2021-11-27

**Authors:** Shucong Xu, Lin Yuan, Lei Wang, Jinyu Li, Fuchang Xu, Zhenzhu Zheng, Debin Shan, Bin Guo

**Affiliations:** 1 National Key Laboratory for Precision Hot Processing of Metals, Harbin 150001, China; xushucong1995@163.com (S.X.); allengineer@163.com (L.W.); LJY02150301@163.com (J.L.); xfch@hit.edu.cn (F.X.); zzzheng@hit.edu.cn (Z.Z.); shandebin@hit.edu.cn (D.S.); guobin@hit.edu.cn (B.G.); 2 School of Materials Science and Engineering, Harbin Institute of Technology, No. 92 Xi Dazhi Street, Harbin 150001, China

**Keywords:** whiskers, aluminum matrix composites, forging, secondary forgeability

## Abstract

The poor formability of high volume fraction whisker reinforced aluminum matrix composites of original squeeze casting is an important factor restricting its further development and application. Currently, there are no reports on the secondary forgeability of aluminum matrix composites of original squeeze casting, although some papers on its first forgeability are published. The secondary forgeability is very important for most metals. This study aims to investigate the secondary forgeability of aluminum matrix composites. In this study, the secondary upsetting experiments of 20 vol% SiCw + Al_18_B_4_O_33_w/2024Al composites, treated by the original squeeze casting and extrusion, were carried out. The first upsetting deformation is close to the forming limit, the secondary upsetting deformation under the same deformation conditions was carried out to investigate the secondary forgeability. The experimental results show that, unlike aluminum alloys, the 20 vol% SiCw + Al_18_B_4_O_33_w/2024Al composites at the original squeeze casting and extrusion states have no secondary forgeability due to the whisker rotating and breaking during the secondary upsetting. The high volume fraction whisker reinforced aluminum matrix composites of original squeeze casting cannot be formed by the multiple-forging method since the cavities and cracks caused by whisker fracture continue to expand during secondary processing, which leads to further extension of macroscopic cracks.

## 1. Introduction

Metal matrix composites (MMCs), especially those based on light metals such as aluminum alloys, have become an indispensable lightweight structural material in high-tech areas such as defense aerospace and military equipment due to its advantages of light mass, high strength, well thermal stability, wear resistance and low thermal expansion coefficient [1,2,3,4,5,6,7,8,9,10,11,12]. However, MMCs have low elongation, poor plastic forming ability and low material utilization, making it difficult to form complex parts, which is the most important reason to limit their application [13,14,15,16,17,18,19].

Aluminum matrix composites have poor forgeability and a narrow forging temperature range. Deformation of composites is affected by matrix, reinforcement and strain rate [20]. During the plastic deformation process, the reinforcement added to the aluminum matrix composite will fracture easily due to its brittleness and hardness. Microcracks will be induced in the matrix, which reduces the thermoplastic deformation ability of the aluminum matrix composite [21].

To reduce the costs in the industrial production, the original squeeze casting is often used to prepare composite materials. Aluminum matrix composite materials prepared by original squeeze casting usually have casting defects such as pinholes, pores, reinforcement aggregates and low interfacial bonding. The stress concentration will be caused by these defects during plastic deformation which will lead to cracking and poor processing ability [22]. Aluminum matrix composites of high volume fraction whisker reinforced often have an elongation of less than 5% at room temperature. Even at high temperatures, the elongation of composites is difficult to increase by common forming processes. The poor formability of aluminum matrix composites has become an important reason for delaying further application of aluminum matrix composites of original squeeze casting [23]. Fu et al. proposed that the same multi-step forming as ordinary metals could be tried to increase the forgeability of high volume fraction whisker reinforced aluminum matrix composites [24]. The research on the secondary forgeability has practical significance for the development of new processing technology of the aluminum matrix composites.

Some relevant research on the first forgeability have been done in recent years. SiCp/6066 aluminum matrix composites with different volume fractions were prepared by Hu et al. [25] using canned hot extrusion, the relationship between SiCp volume fraction and tensile strength as well as yield strength was studied. The results show that as the volume fraction of the reinforcement increases, the strength of the composite material is improved. When the volume fraction of SiCp is greater than 12%, the strength of the material decreases. A hot extrusion deformation at the extrusion ratio of 25:1 of SiCp/Al with a particle volume content of 25% were performed by Qu et al. [26] who found that hot extrusion deformation can greatly improve the strength, modulus and plasticity of composites. Compared with the base alloy, Zhang et al. [27] found that the elongation of hybrid reinforced composites such as SiCw·SiCp/2024Al decreases, the hot extrusion is beneficial to improve the elongation of hybrid composites. Xu et al. [28] found that the partial melting and occurrence of dynamic recrystallization at high temperature and high strain rate could effectively avoid the formation of cracks and improve the hot workability of 20vol% SiCw/6061 aluminum matrix composites. Yuan et al. [29] prepared the aluminum matrix composites reinforced by aluminum borate whisker with or without Bi_2_O_3_ coating by squeeze casting. It was found that ABOw/Bi_2_O_3_/Al composites have high plastic forming properties. During the preparation process, Bi_2_O_3_ coating react with aluminum matrix in the composite, which forms Bi phase at the interface between whisker and matrix. The fracture of the whisker is obviously reduced and the forming property of the composite is improved. Leng et al. [30] added graphite with different volume fractions and particle sizes, SiC/Gr/Al composites were prepared by squeeze casting, the results show that the elastic modulus of the composites tends to decrease with the increasing of volume fraction and particle size of graphite. Both the tensile strength and the elastic modulus depended on the volume fraction and the particle sizes of graphite. However, only single pass forgeability study of aluminum-based composites has been carried out, whether there is no relevant research on the secondary forgeability of the aluminum matrix composites reinforced with high volume fraction whiskers. The secondary forgeability is very important for most of metals. Therefore, the research on secondary forgeability of aluminum matrix composites has important practical significance.

This paper studies the forging deformation behavior of 20 vol% SiCw + Al_18_B_4_O_33_w/2024Al composites, explores the secondary forgeability of whisker-reinforced 20 vol% SiCw + Al_18_B_4_O_33_w/2024Al composites of original squeeze casting, and provides a reference for the design of the thermoplastic forming process for high volume fraction whisker-reinforced aluminum matrix composites of original squeeze casting.

## 2. Materials and Methods

### 2.1. Materials

The material used in the experiment is 20 vol% SiCw + Al_18_B_4_O_33_w/2024Al prepared by original squeeze casting. The chemical composition of SiCw + Al_18_B_4_O_33_w/2024Al is shown in Table 1. The total volume fraction of the whisker reinforcement is 20%, and the ratio of SiCw to Al_18_B_4_O_33_w is 1:4. The SiC whisker is β-SiC with a diameter of 0.1–1.0 μm and a length of 10–50 μm, the Al_18_B_4_O_33_w whisker has a diameter of 0.5–1.0 μm and a length of 10–30 μm, the matrix is the 2024 aluminum alloy. The material was prepared by secondary pressure processing, including low-pressure infiltration and high-pressure holding and cooling. The preformed block and the mold were preheated to 500–520 ℃ and the heat was preserved, while the aluminum alloy was heated to 700–800 ℃ to melt and maintain the temperature. Then, the aluminum alloy was poured into the preheated mold, and the indenter pressurized the aluminum alloy in the molten state at a speed of 2 mm/s, and holding the pressure at 50–60 MPa for 10 min.

### 2.2. Methods

#### 2.2.1. Extrusion Experiment

**Figure 1:**
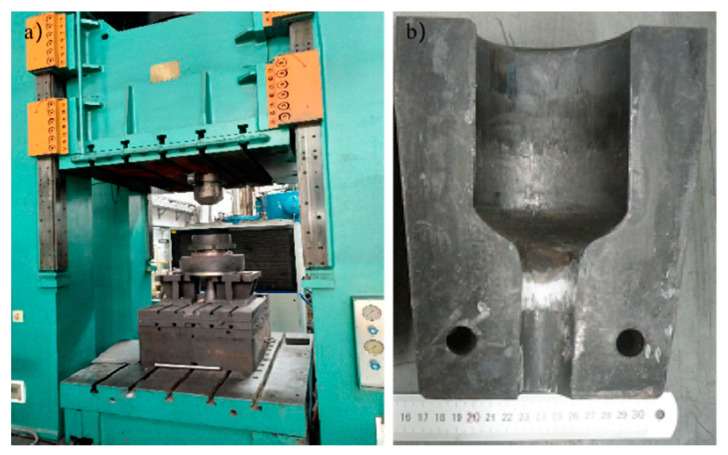
Hydraulic press and die used in extrusion experiments: (**a**) The 1600 t hydraulic press; (**b**) The extrusion die.

The extrusion test was performed, and the research on secondary forgeability of extruded aluminum matrix composites can be carried out. The extrusion experiment of SiCw + Al_18_B_4_O_33_w/2024Al composite material was carried out using a 1600 t hydraulic press, the hydraulic press and extrusion die are shown in Figure 1. The diameter of the upper part of the extrusion die cavity is φ74 mm, the depth is 100 mm, the central cone angle is 120°, the arc boundary transition is 10 mm, the extrusion diameter is 25 mm and the extrusion ratio is 9:1. The size of the original billet is φ72 mm× 60 mm. Heating the billet and the extrusion die with a heating furnace, the billet was taken out when it was heated about 10 min, the graphite lubricant was brushed on the surface. Then, it was placed in the heating furnace, heated to the specified temperature and held for 2 h. After the extruding, the billet was cooled in air.

#### 2.2.2. Upsetting Experiment

The upsetting tests were performed on composites of the original squeeze casting and extrusion. Two sizes of samples were used in the upsetting tests. The distribution of the reinforcing phase of the original squeeze casting is uneven, so large samples with the size of 60 mm × 60 mm × 60 mm were used for the upsetting test. Given the extrusion composite material has been deformed, the distribution of the reinforcing phase is uniform, and the forgeability is improved, there is no need to use large samples, so small samples with the size of 14 mm × 14 mm × 14 mm were used for the upsetting test.

The upsetting test was performed on samples with a size of 14 mm × 14 mm × 14 mm under the AG-X Plus electronic universal testing machine. The samples were placed in the test machine and heat preserved for 30 min. After upsetting in the axial direction, quenching the compressed composites in cold water to retain the microstructure. The upsetting test was performed on samples with a size of 60 mm × 60 mm × 60 mm under a 5000 t press. The samples were heated to the specified temperature and heat preserved for 1 h. Upsetting the samples took place in the direction of pressure during the squeeze casting, then the compressed composites were quenched in cold water to retain the microstructure.

## 3. Results

### 3.1. The Sheathed Extrusion of the Aluminum Matrix Composites of Original Squeeze Casting Samples

**Figure 2:**
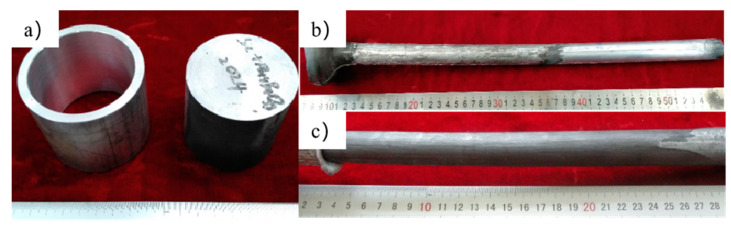
The billet and extruded bars of sheathed extrusion: (**a**) The billet of aluminum matrix composite with the size of φ60 mm × 60 mm and the pure aluminum sheath; (**b**) The surface of SiCw + Al_18_B_4_O_33_w/2024Al composite extruded bars by sheathed extrusion; (**c**) The surface of SiCw + Al_18_B_4_O_33_w/2024Al composite extruded bars by sheathed extrusion after removing the sheath.

The sheath was a pure aluminum ring with the outer diameter of φ72 mm, the inner diameter of φ60 mm and the height of 60 mm. The size of the billets of aluminum-based composite material is φ60 mm × 60 mm. The billet and extruded bars are shown in Figure 2.

The experiment results show that although there are few transverse cracks on the sheath, the surface of SiCw + Al_18_B_4_O_33_w/2024Al composite has no macro cracks after removing the sheath, indicating that the forming quality of the composite is excellent. This is because that the pure aluminum sheath avoids the direct contact of the composite and the extrusion die, which reduces the surface friction. In addition, the sheath also has a thermal insulation effect on the aluminum-based composite material, which makes the internal aluminum matrix composite cool down more slowly, improving the forming ability of the aluminum matrix composites.

### 3.2. The Secondary Forgeability of the Aluminum Matrix Composites of Original Squeeze Casting Samples

#### 3.2.1. The First Forging Limit of the Original Squeeze Casting Samples

**Figure 3:**
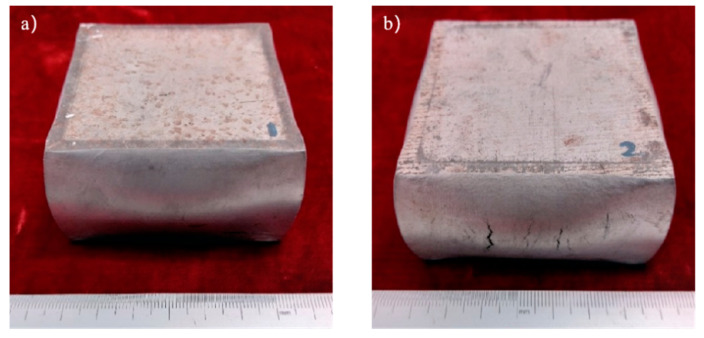
The samples morphology of the aluminum matrix composite: (**a**) Compression deformation amount of 32.43%; (**b**) Compression deformation amount of 34.28%.

The sample morphology of the aluminum matrix composite after the upsetting test is shown in Figure 3. When the axial compression deformation amount was 32.43%, the sample height after compression was 40.30 mm. There were no macroscopic cracks on the surface of the sample. When the axial compression deformation amount was 34.28%, the sample height after compression was 39.43 mm. Deep macro cracks appeared on the surface of the sample, and the direction of the cracks were parallel to the compression direction. The first forging limit of the aluminum matrix composite of original squeeze casting is 32.43%.

#### 3.2.2. The Secondary Upsetting Experiment of the Original Squeeze Casting Samples

The upsetting experiments of the SiCw + Al_18_B_4_O_33_w/2024Al composite samples of the original squeeze casting were carried out, as shown in Table 2.

It can be observed from the figure that after the secondary upsetting experiments, it was easy to induce macro cracks on the surface of the samples. When the secondary deformation amount was 5.17%, there were no macro cracks on the surface, but the total axial deformation did not exceed the forging limit of 32.43%. In conclusion, the accumulative deformation of the original squeeze casting after two times upsetting experiments does not exceed the forming limit of one time upsetting.

### 3.3. The Secondary Forgeability of the Aluminum Matrix Composites of Extrusion Samples

#### 3.3.1. The First Forging Limit of the Extrusion Samples

**Figure 4:**
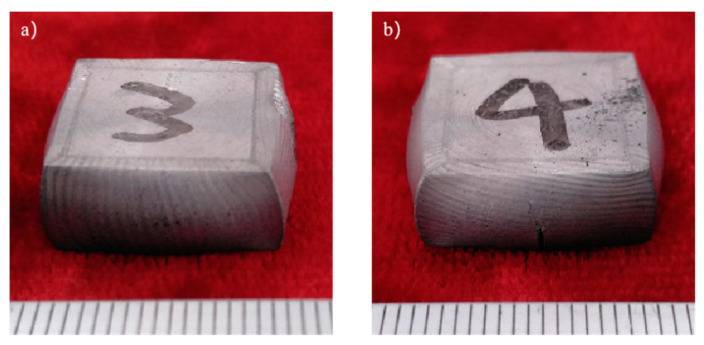
The samples morphology of the aluminum matrix composite: (**a**) Compression deformation amount of 46%; (**b**) Compression deformation amount of 50%.

The samples morphology of the aluminum matrix composite after upsetting test is shown in Figure 4. When the axial compression deformation amount was 46%, the surface of the sample had no macroscopic cracks. When the axial compression deformation amount was 50%, there were only shallow cracks on the surface of the samples. The forming limit of the SiCw + Al_18_B_4_O_33_w/2024Al composite of extrusion is 46%. The aluminum matrix composite of extrusion has better forgeability than the original squeeze casting.

#### 3.3.2. The Secondary Upsetting Experiment of the Extrusion Samples

The upsetting experiments of the SiCw + Al_18_B_4_O_33_w/2024Al composite samples of extrusion were carried out, as shown in Table 3.

It can be observed from the figure that after the secondary upsetting experiment, micro-cracks appeared on the surface of the samples. The total axial deformation did not exceed the forging limit of 46%. In conclusion, the accumulative deformation of the extrusion after two times upsetting experiments does not exceed the forming limit of one time upsetting.

## 4. Discussion

### 4.1. Microstructure of the Original Squeeze Casting Samples

**Figure 5:**
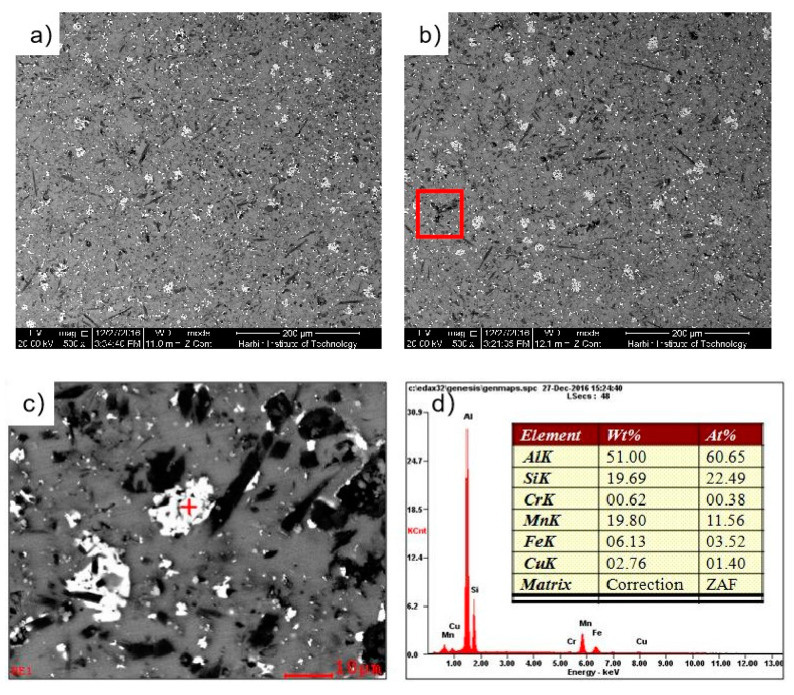
The SEM photograph and second phase energy spectrum of the microstructrue of the casting composite: (**a**) The horizontal microstructrue of SEM; (**b**) The vertical microstructrue of SEM; (**c**) The large second phase in matrix; (**d**) The energy spectrum of large second phase.

The SEM photographs of the SiCw + Al_18_B_4_O_33_w/2024Al matrix composite of the original squeeze casting are shown in Figure 5. The whisker reinforcements are randomly oriented and dispersively distributed in the aluminum matrix, but there are also some partial whisker aggregations, as shown in the red square of Figure 5b. This is because that the molten aluminum alloy cannot fully penetrate into the gap between the whisker reinforcements during the preparation process of squeeze casting, which results in many defects in the casting composite. These defects are easy to induce stress concentration and micro-cracks during deformation, which reduces the deformation ability of the composite. The second phases with different sizes are dispersively distributed in the aluminum matrix. The result of energy spectrum analysis shows that the main components were Al, Si, Mn, etc., the chemical formula of the second phase infers as Al_5_Si_2_Mn. The casting defects, unevenly distributed whiskers and brittle second phases result poor forgeability of the aluminum matrix composites.

### 4.2. Microstructure of the Extrusion Samples

**Figure 6:**
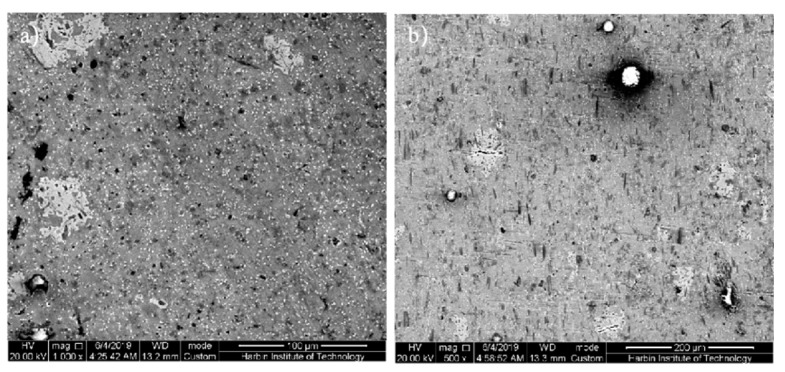
The SEM photograph of the microstructure of the extruded composite: (**a**) Transverse section; (**b**) Axial section.

The SEM photographs of the SiCw + Al_18_B_4_O_33_w/2024Al matrix composite of sheathed extrusion are shown in Figure 6. Compared with the random distribution of the whiskers of original squeeze casting, most whiskers are parallel to the extrusion direction, and only a few whiskers are perpendicular to the extrusion direction. This is because the whiskers rotate during extrusion, the orientation of the whiskers is parallel to the extrusion direction and the whiskers are uniformly distributed in the aluminum matrix. When the extruded composite is axially forced, the reinforced whiskers distributed along the axial direction can most effectively withstand the load transmitted from the matrix. For the original squeeze casting composites, since the wetting angle of SiC on Al can be as high as 155°, the molten aluminum alloy with high surface tension is difficult to fill the area of whiskers aggregation. The large number of pores in the matrix alloy not only reduce the density of the aluminum matrix composite, but also easily induce stress concentration during thermoplastic deformation. As there is no matrix support in the whisker aggregation area, the whiskers are likely to fracture during plastic deformation, and micro cracks are induced inside the composite material, these micro-cracks are easily to propagate and form macro cracks. The matrix alloy is under three-dimensional compressive stress during the extrusion process, which can effectively prevent the generation of crack sources and the propagation of cracks and eliminate the squeeze casting defects such as pores and pinholes in the matrix alloy. The porosity decreases, the density increases and the reinforcements are uniformly distributed after extrusion, which make the strength of the matrix alloy increase and achieve better a microstructure and better properties, and, furthermore, create favorable conditions for the secondary forming. The coarse grains of squeeze casting can be refined during extrusion, but there are still some large-size pores that are not eliminated as shown in Figure 6a, the bulk second phases in the whiskers aggregation area are easily to break into micro cracks, which can induce cracking.

### 4.3. Fracture Mechanism of the Aluminum Matrix Composite of the Original Squeeze Casting and Extrusion

The SiC whisker with excellent chemical stability has no interface chemical reaction with the aluminum matrix, the interface bonding between the aluminum alloy and the whiskers is mainly mechanically physics bonding. Whisker debonding from the matrix is mainly due to the low interface bonding strength. The load is transferred from the matrix to the interface. If the interface bonding strength is higher than the whisker and matrix strength, whisker fracture or matrix interface cracking occurs preferentially. If the interface bonding strength is lower than the whisker and matrix strength, the whiskers detach from the bonding interface and are pulled out from matrix.

**Figure 7:**
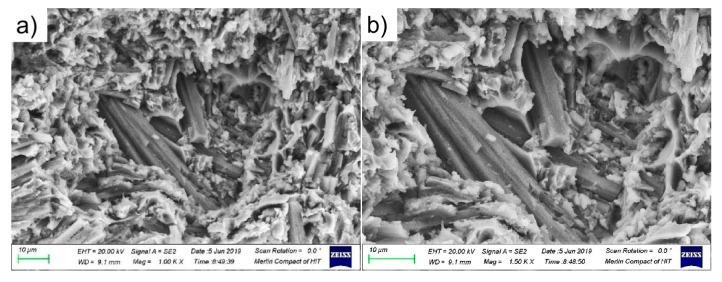
The high-temperature tensile fracture at 450 °C of the original squeeze casting: (**a**) 1000 times; (**b**) 1500 times.

The high-temperature tensile fracture at 450 °C of the aluminum matrix composite of original squeeze casting is shown in Figure 7. The analysis of the tensile fracture surfaces reveal that whiskers fracture mostly occurs on the fracture surface, the whiskers are rarely pulled out from the matrix alloys and there are no obvious separation between the whiskers and the matrix alloys. The orientation of the whisker fracture is often different from the tensile direction, the whiskers tend to rotate or break in the tensile direction. Due to the disordered orientation of the whiskers of the original squeeze casting, there are many whiskers perpendicular to the tensile direction and the whiskers break easily during tension, which is the main reason of cracking of aluminum matrix composites. The high-temperature tension fracture of the aluminum matrix composites of original squeeze casting is induced by whisker fracture.

**Figure 8:**
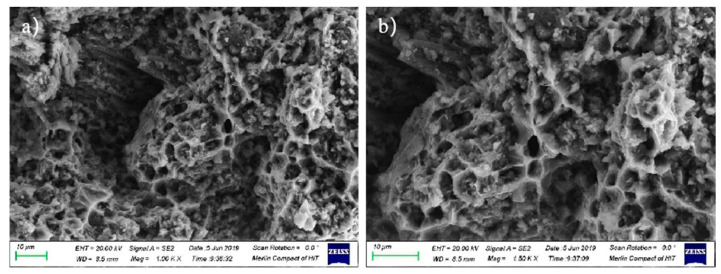
The high-temperature tensile fracture at 450 °C of the extrusion: (**a**) 1000 times; (**b**) 1500 times.

The high-temperature tensile fracture at 450 °C of the aluminum matrix composite of extrusion is shown in Figure 8. A large number of pits can be observed, which were left by the whiskers pulled out from the matrix. The defects of the squeeze casting such as pores are reduced after sheathed extrusion, and the whiskers are oriented along the extrusion direction. The whiskers in the original squeeze casting are randomly distributed, rotate in the direction of tension during the tensile deformation and are easy to crack in this process. However, the randomly distributed whiskers will bear the shear force in the tensile direction, which increases the strength of aluminum matrix composites. Therefore, the tensile strength is affected by two aspects of whisker orientation. Different from the original squeeze casting, the orientation of the whiskers in the extrusion are uniformly distributed in the direction of extrusion, the orientation of the whiskers is consistent with the tensile direction during the tensile deformation, the whiskers rarely rotate, and the load is transferred from the matrix to the interface. Due to the low bonding strength of the whiskers and the matrix interface, most of the whiskers are pulled out from the interface. The high-temperature tensile strength and elongation of the extruded aluminum matrix composite are mostly affected by the interface strength, the interface between the matrix and whiskers is smooth and there is no chemical reaction at the interface. The bonding between the matrix and the whisker at the interface is mainly mechanically physics bonding, so the interface combining ability is weak.

The SiCw + Al_18_B_4_O_33_w/2024Al composites are whisker-reinforced composites. According to the shear lag model in mechanics of short fiber composites, the critical aspect ratio of whiskers in the whisker-reinforced aluminum matrix composite is shown in Equation (1).

(1) $$\frac{Lc}{d}=\frac{\sigma_{ff}}{4\tau_{b}}$$

In this equation:

$\sigma_{ff}$ is the breaking strength of the whiskers,

$\tau_{b}$ is the shear strength of the interface,

$L_{c}$ is the length of the whiskers,

d is the diameter of the whiskers.

When the aspect ratio of the whiskers of the aluminum matrix composites is higher than the critical aspect ratio, the whiskers can withstand the load from the matrix, the pullout of the whiskers is the main form of fracture at this time. The aspect ratio of the SiCw + Al_18_B_4_O_33_w/2024Al composites prepared by squeeze casting is constant, and the breaking strength of the whiskers is determined by the whisker materials. The whisker breaking strength is a constant value. From the definition equation of critical aspect ratio, it can be found that the critical aspect ratio of whiskers of SiCw + Al_18_B_4_O_33_w/2024Al composites is inversely proportional to the shear strength of the matrix interface, the higher the strength of the matrix, the lower the critical aspect ratio of the whiskers of the aluminum matrix composite. After sheathed extruding the SiCw + Al_18_B_4_O_33_w/2024Al composite of the original squeeze casting, the whiskers rotate because of the partial nonuniform plastic flow, the whiskers fracture under severe three-dimensional compressive stress. Therefore, the aspect ratio of the whisker of the extruded aluminum matrix composite is smaller than that of the original squeeze casting. Compared with the extrusion, there are a larger number of whiskers exceeding the critical aspect ratio in the original squeeze casting, which results in more whiskers of the original squeeze casting being loaded. Thus, in the tensile fracture of the aluminum matrix composites of original squeeze casting, the fracture of the whiskers exceeding critical aspect ratio is the main fracture form, while in the tensile fracture of the extruded aluminum matrix composite, the pullout of the whiskers below the critical aspect ratio is the main fracture form.

### 4.4. Microstructure of the Original Squeeze Casting Samples after Secondary Upsetting

Compared with the original squeeze casting, the orientation of the whiskers inside the material changes significantly after upsetting to the forming limit of the aluminum matrix composite.

**Figure 9:**
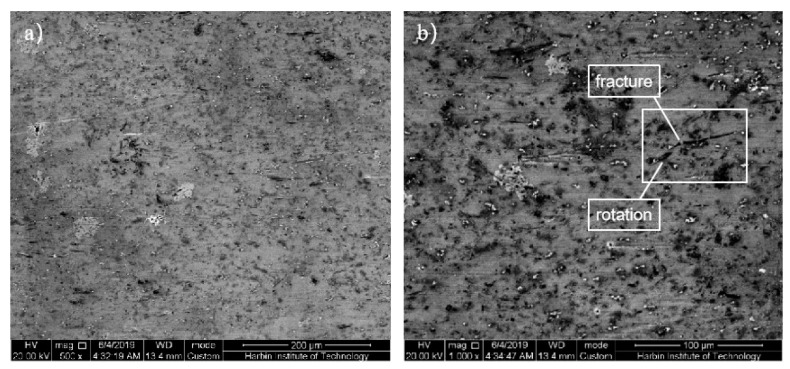
The SEM microstructure of samples after the first upsetting deformation: (**a**) 500 times; (**b**) 1000 times.

**Figure 10:**
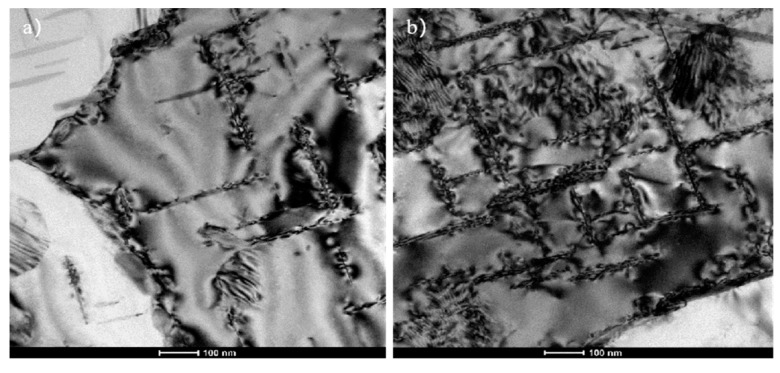

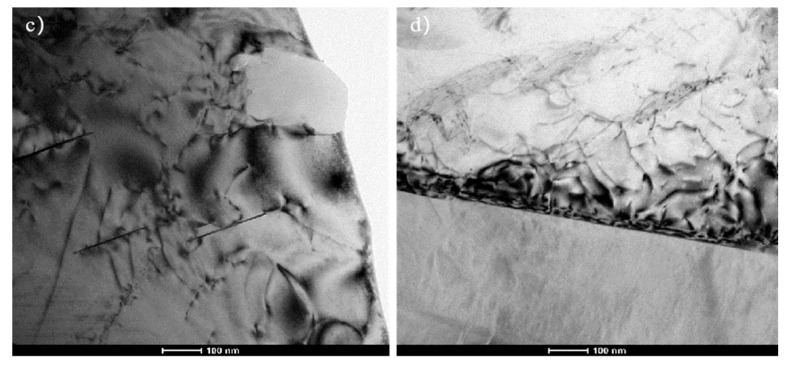
Accumulation of the dislocations at the interface: (**a**) Accumulation of the dislocations at grain boundaries; (**b**) Accumulation of the dislocations at the interface of the second phase; (**c**) Accumulation of the dislocations at the second phase after heat preserving; (**d**) Accumulation of the second deformation dislocations at the Al_18_B_4_O_33_w interface.

After the first upsetting deformation, the whiskers rotate and break, as shown in Figure 9. The work hardening occurs inside the matrix due to plastic deformation and a large amount of dislocation accumulates at the interface. As shown in Figure 10a,b,a large amount of dislocations are accumulated at the grain boundary and the needle-shaped second phase. The samples were heat preserved at 480 ℃, the holding time is consistent to that of the first upsetting and the dislocation density decreases after recovery of the matrix. The dislocation density at the second phase is significantly reduced, as shown in Figure 10c, and the work hardening effect of the matrix is reduced. However, the whiskers continue rotate during the second upsetting deformation, the nonuniform flow of metal in the matrix causes the whiskers to withstand bending torque and dislocations are accumulated at the interface, which makes the whiskers continue to break during rotation. The Al_18_B_4_O_33_w is accumulated at the interface as shown in Figure 10d. In the process of rotating and breaking of the whiskers, the dislocation density of the matrix decreases after recovery, and the flow ability of the matrix decreases due to work hardening, and the dislocations at the whisker interface are reduced, which is conducive to increase the secondary deformation amount. Due to the recovery softening of matrix metal, the deformation resistance is reduced, the flow ability of the matrix is relatively improved and the difference of the partial deformation rates are also increased. In addition, due to the decrease of the deformation resistance of the matrix, the proportion of the loading on whiskers increases, which makes the whiskers easier to rotate and break. The softening of matrix metal affects the secondary forgeability in two aspects. For the aluminum matrix composite, the whisker fracture has a greater effect on reducing plasticity, so it does not exhibit secondary forgeability similar to traditional metal.

### 4.5. Microstructure of the Extrusion Samples after Secondary Upsetting

**Figure 11:**
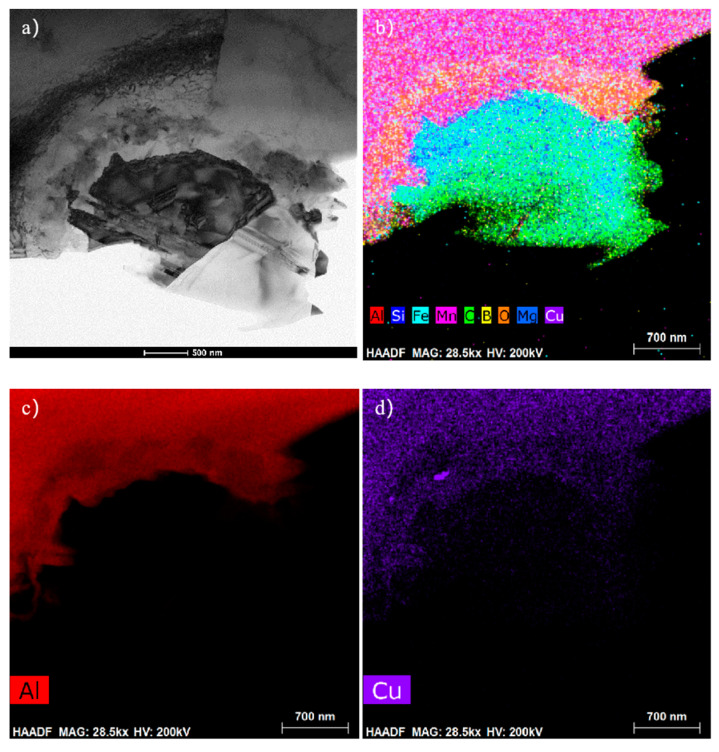

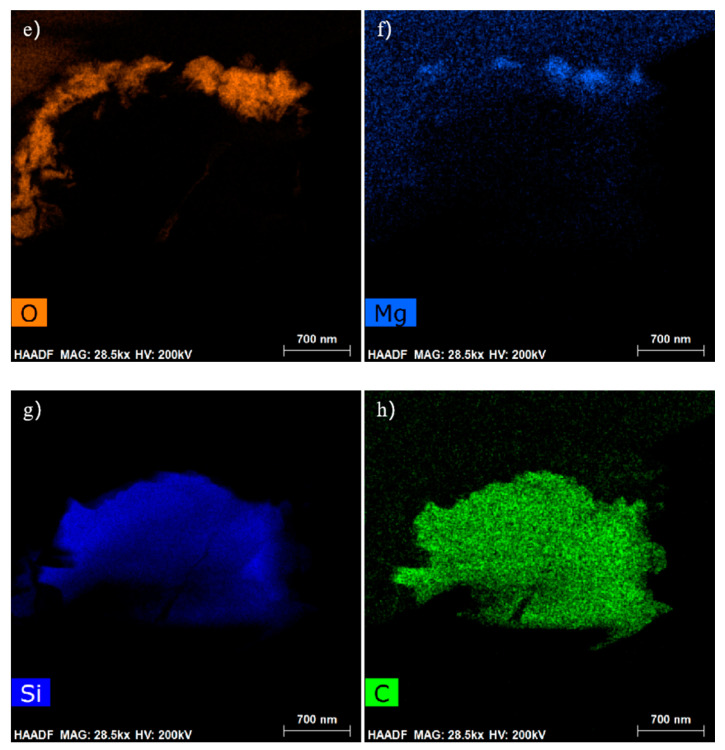
The transmission map scanning after compression: (**a**) The bright-field image; (**b**) The distribution of all elements, The element distribution of (**c**) Al; (**d**) Cu; (**e**) O; (**f**) Mg; (**g**) Si; (**h**) C.

The transmission bright field image of the extruded aluminum matrix composite is shown in Figure 11. There are a large number of dislocations at the whisker interface, which form dislocation walls. According to the map scanning analysis of each element, the distribution of whiskers and matrix can be visually observed. The distribution of the coincidence of Si and C elements can reflect the distribution of SiC whiskers. The distribution of Cu and Al indicates that Cu segregates in the form of CuAl_2_ on the surface of SiC whiskers, O is enriched at the interface of the outer layer of CuAl_2_ and formed an Al_2_O_3_ layer with a thickness of 200 nm to 400 nm. The SiC whisker interface is flat, there is no chemical reaction with the matrix and no interface reactants are observed at the interface.

**Figure 12:**
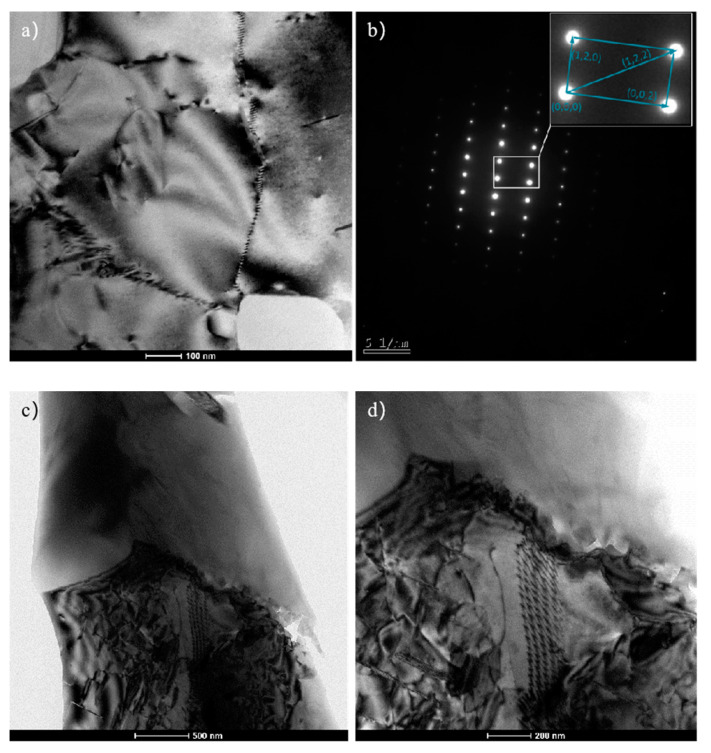
Recovery and whisker fracture of the extruded aluminum matrix composites: (**a**) The sub-grain boundary; (**b**) Al_18_B_4_O_33_w diffraction spots; (**c**) The whisker fracture; (**d**) The interface at the fracture of the whisker.

Compared to the random orientation and dispersive distribution of the whiskers of the original squeeze casting, the orientation and distribution of whiskers have changed greatly, the decrease in the aspect ratio of the whiskers and the distribution of the whiskers change the fracture mechanism of high-temperature tensile after extrusion and whisker pullout is the main form of fracture. This means that the extruded aluminum matrix composite has better whisker orientation and distribution which protects the whiskers from breaking during tensile deformation. Although the orientation of the whiskers of the extruded aluminum matrix composite is parallel to the extrusion direction, which is beneficial to the tensile deformation, the secondary upsetting is different from the tensile deformation. After the first upsetting deformation and heat preservation, the matrix generates sub-grain boundaries during recovery, as shown in Figure 12a. Dislocations in the matrix are greatly reduced, and the matrix is softened, but the whiskers have already rotated after the first upsetting and the angle between the whiskers and extrusion direction is 29°. The whiskers still rotate and break during the secondary upsetting, as shown in Figure 12c,d.

**Figure 13:**
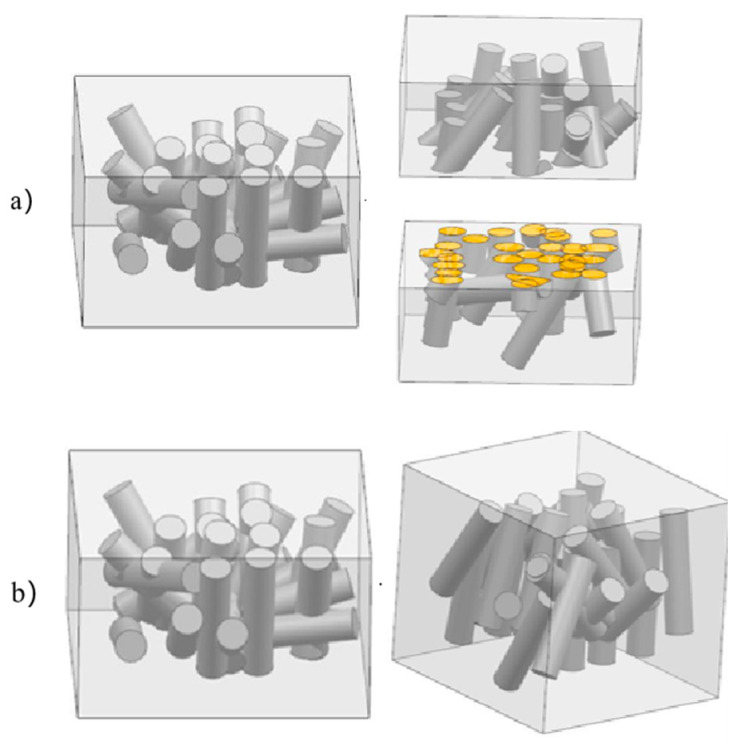
The schematic diagrams of the deformation of the original squeeze casting: (**a**) Aluminum matrix composites during tension; (**b**) Aluminum matrix composites during upsetting.

For the aluminum matrix composite of original squeeze casting, the internal whiskers are randomly distributed. During tension, the whiskers with disorderly orientation and distribution rotate in tensile direction due to tensile stress. The whiskers in the vertical tensile direction withstand shear stress, which commonly makes the whiskers break during tensile, as shown in Figure 13a. The disorderly whiskers are easily broken by partial nonuniform flow stress during upsetting, as shown in Figure 13b, which limits the aluminum matrix composites to exhibit secondary forgeability.

**Figure 14:**
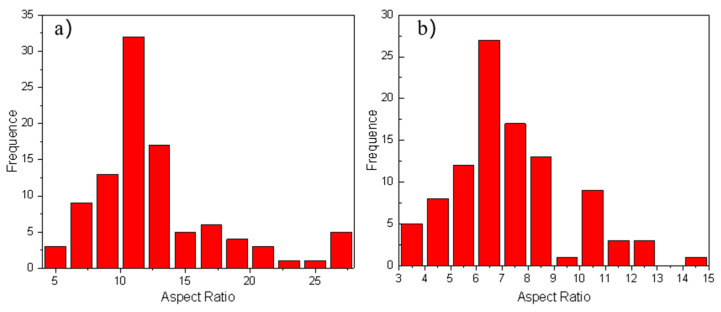
The change of the aspect ratio after extrusion: (**a**) The original squeeze casting, (**b**) The extrusion.

**Figure 15:**
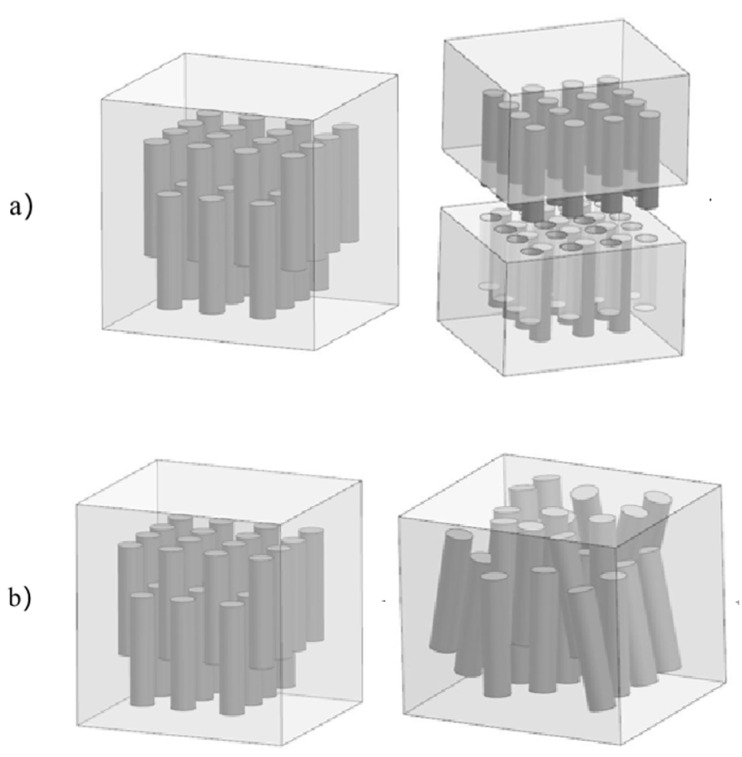
The schematic diagrams of the deformation of the extrusion: (**a**) Aluminum matrix composites during tension; (**b**) Aluminum matrix composites during upsetting.

The aspect ratio of the whiskers of aluminum matrix composite was measured by SEM Photographs with high magnification, as shown in Figure 14. It can be seen that the aspect ratio decreases after extrusion due to the fracture of part of the whiskers. As a result of the specific orientation and the decrease of the aspect ratio of the whiskers, a large number of whiskers are under uniform stress field during tension and only a small part of the whiskers rotate or break, which results in better plastic deformation ability and higher elongation of the aluminum matrix composite, as shown in Figure 15. Although the extruded aluminum matrix composites show higher elongation in tensile test, the direction of the whiskers parallel to the extrusion direction changes due to the deformation of the matrix during the first upsetting deformation, leading to some whiskers rotating and breaking in the upsetting deformation. The cracks in the aluminum matrix composites reinforced with high volume fraction whiskers are easily propagated, thus the extruded aluminum matrix composites do not have the secondary forgeability due to whisker rotation fracture.

## 5. Conclusions

In the study of the secondary forgeability of the 20 vol% SiCw + Al_18_B_4_O_33_w/2024Al whisker reinforced composite using the original squeeze casting and extruded samples, the results can be outlined as follows:

(1) The surface forming quality of the extruded bars is different at different positions, and the extruded bars are easy to induce macro cracks. Bars with better forming surface quality can be obtained by using sheathed extrusion, and the extrusion forming quality of aluminum matrix composite can be improved. Although there are few transverse cracks on the sheath, the surface of SiCw + Al_18_B_4_O_33_w/2024Al matrix composite has no macro cracks after removing the sheath, indicating that the forming quality of the composite is excellent. The porosity decreases, the density increases and the reinforcements are uniformly distributed after extrusion, which make the strength of the matrix alloy increase and achieve better microstructure and properties.

(2) The upsetting test of aluminum matrix composites of the original squeeze casting and extrusion was carried out to determine the forming limit. The forming limit of the original squeeze casting composite is 32.43%, and that of extruded composite is 46%. The accumulative deformation after two times upsetting experiments does not exceed the forming limit of one time upsetting. The results show that 20 vol% SiCw + Al_18_B_4_O_33_w/2024Al whisker reinforced composites of the original squeeze casting and extrusion do not has the secondary forgeability. This is not similar to traditional metals.

(3) The SiCw + Al_18_B_4_O_33_w/2024Al whisker reinforced composites of the original squeeze casting and extrusion do not has the secondary forgeability. Although the aluminum matrix still has recovery ability, and the matrix dislocation density decreases during the heat preservation, the whiskers are easy to rotate and fracture, and the pores and cracks formed by whisker fracture continue to expand during the secondary deformation, which leads to further propagation of macroscopic cracks. This means that it is difficult to form complex forgings of high volume fraction whisker reinforced aluminum matrix composite of the original squeeze casting by traditional forging methods, even though strict isothermal forging methods.

## Figures and Tables

**Table 1:** Chemical composition of 2024 aluminum alloy (mass fraction wt.%).

Si	Fe	Cu	Mn	Mg	Zn	Ti	Cr	Al
0.5	0.5	3.5–4.9	0.3–0.9	1.2–1.8	0.25	0.15	0.10	other

**Table 2:** The secondary upsetting morphology of original squeeze casting composites.

**No.**	**Morphology of the Samples**	**Forging Process**	**Surface Crack**
1	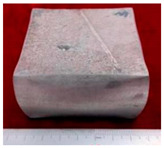	Axial compression 26.83% Sample height after deformation 43.90 mm	No macroscopic cracks on the surface
2	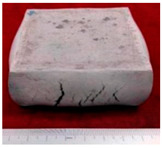	Axial compression of 26.83% 480 °C for 1 h Axial compression 14.84% Total axial deformation 41.67% Sample height after deformation 43.90 mm	Severe macroscopic cracks on the surface
3	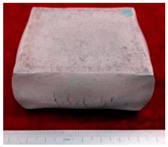	Axial compression 26.83% 480 °C for 1 h Axial compression 10.33% Total axial deformation 37.05% Sample height after deformation 37.70 mm	Shallow macroscopic cracks on the surface
4	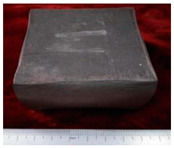	Axial compression 26.83% 480 °C for 1 h Axial compression 5.17% Total axial deformation 32% Sample height after deformation 40.80 mm	No macroscopic cracks on the surface

**Table 3:** The secondary upsetting morphology of extruded composites.

**No.**	**Morphology of the Samples**	**Forging Process**	**Surface Crack**
1	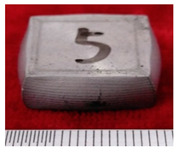	Axial compression 37.86% 480 °C for 30 min Axial compression 6.42% Total deformation 44.28% Sample height after compression 7.8 mm	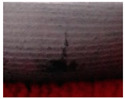 Micro-cracks on the surface
2	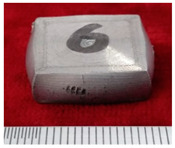	Axial compression 37.86% 480 °C for 30 min Axial compression 7.1% Total deformation 45% Sample height after compression 7.7 mm	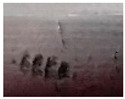 Micro-cracks on the surface
3	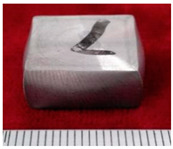	Axial compression 37.86% 480 °C for 30 min Sample height after compression 8.7 mm	No macroscopic cracks on the surface

## Data Availability

Data sharing not applicable.
